# Glomus Tumors of the Hand

**Published:** 2008-10-08

**Authors:** Ron Hazani, John M. Houle, Morton L. Kasdan, Bradon J. Wilhelmi

**Affiliations:** Division of Plastic Surgery, University of Louisville School of Medicine, Louisville, KY

## Abstract

**Objective:** The *purpose* of this study is to present a review of the current understanding of glomus tumors of the hand. **Methods:** Clinical cases are used to demonstrate the relevance of history and physical examination in deriving the diagnosis of this rare, but important entity. Treatment, complications, and review of the literature are presented. **Results:** Glomus tumors are rare vascular lesions representing approximately 1% of all hand tumors. Derived from the glomus body, they are usually found at the tip of digits and present as a classic triad of severe pain, point tenderness, and cold sensitivity. Clinical features include blue discoloration, palpable nodule, and nail deformity in subungual tumors. The Hildreth's test and the Love's pin test are reliable methods of diagnosing glomus hand tumors with sensitivity and specificity exceeding 90%. Surgical excision is the treatment of choice. Possible complications following operative management include recurrence and nail deformity. **Conclusion:** This article outlines the current knowledge relating to the pathophysiology, diagnosis, and treatment of glomus tumors of the hand.

Masson first described glomus tumors in 1924.^1^ These are benign, vascular hamartomatous derivatives of the glomus body—a normal intradermal arteriovenous anastomosis that arises from the normal neuromyoarterial glomus. These structures regulate skin temperature.^2^ They are present in the tips of the digits, particularly in the subungual area.^3^ The glomus body consists of an arteriole, a venule, and an anastomotic vessel without an intervening capillary bed.^4^ Histologic findings include endothelial pericytes and numerous nonmyelinated nerve fibers.^2^

Glomus tumors account for approximately 1% of all hand tumors^5^ and are even less common in children. Colon and Upton^6^ reported a series of 349 pediatric hand tumors, 9 of which were glomus tumors. Glomus tumors are more common in women between 30 and 50 years of age, are not known to be associated with any other condition, and occur spontaneously. They are usually solitary but a multiple glomus tumor syndrome has been described that is transmitted in an autosomal dominant pattern.^5^

Glomus tumors present as a classic triad of severe pain, point tenderness, and cold sensitivity^1^,^3^,^4^ In a series of 51 patients with glomus tumors of the hand, by Van Geertruyden et al,^1^ spontaneous pain was seen in 80% of patients. Sensitivity to touch was present in 100% of patients and cold sensitivity in 63%. Bhaskaranand and Navadgi^4^ reported, in their series of 18 hand tumors, that of the 14 patients with glomus tumors all presented with severe pain. The 4 patients with other types of tumors presented with dull, aching pain. In the series, 100% of patients had point tenderness and 78% had cold sensitivity. Carroll and Berman^7^ state that excruciating, paroxysmal pain is pathognomonic for glomus tumor.

*Clinical features* of glomus tumors include nail deformity (Fig 1), blue discoloration (Fig 2), and palpable nodule. Van Geertruyden et al^1^ reported rates of 27%, 29%, and 6% for these findings, respectively. When only dorsal tumors are considered, the incidence of nail deformation rises to 47% and blue discoloration is seen in 43% of such lesions.

## DIAGNOSIS

Diagnosis of glomus tumors is primarily clinical. Several clinical tests are useful for diagnosing glomus tumors. Love^8^ reported that localization of the tenderness to an area and the size of a pinhead was suggestive of glomus tumor. For a positive Love's pin test, the patient should experience severe pain and reduction in pain when the skin overlying the tumor is pressed with a pinhead, ballpoint pen, end of a paperclip, or Kirschner wire. The Love test is 100% sensitive and specific according to Bhaskaranand and Navadgi's series.^4^ The cold-sensitivity test is positive when immersing the hand in cold water elicits severe pain in and around the lesion. In addition, there should be a history of cold weather aggravating the symptoms. Hildreth's test is another reliable clinical sign for the diagnosis of glomus tumors.^9^ This test is performed by elevating the patients' arm to exsanguinate it. A tourniquet is inflated to 250 mm~Hg and the tumor is palpated, the pain and tenderness should be reduced. A test is positive when releasing the cuff causes a sudden onset of pain and tenderness in the area of the tumor. In his series of 24 patients with hand tumors, Giele noted a positive Hildreth's test in 13 patients. Twelve of these had glomus tumors and 1 had a hemangiopericytoma with a sensitivity of 92% and specificity of 91%.

Radiography, ultrasound, magnetic resonance imaging, and angiography, all have been used with variable amounts of success for diagnosis and localization of lesions.^1^,^3^,^10^,^11^

## TREATMENT

**Figure 3:**
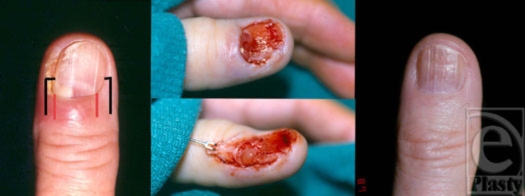
A 67-year-old woman with subungual glomus tumor of the right thumb. Preoperative view of the lesion. Black lines represent the lateral incisions through the paronychium. Red lines represent placement of incisions that may damage the hyponychium (left); view of the nail bed after nail plate removal (center, above); and intraoperative view of the tumor (center, below); and one-year follow-up demonstrating a healed nail bed (right).

Surgical excision is the treatment for glomus tumors; no medical therapy exists.^5^ Excision of the tumor results in resolution of symptoms in all cases.^1^,^3^,^4^ Love's test is used to localize the tumor and it is completely excised under direct vision. Subungual tumors are approached transungually by removing the nail plate and incising the nail bed longitudinally. For lesions that are deep-seated proximally, lateral incisions through the paronychium are recommended. This approach can facilitate better access to the germinal matrix (Fig 3), whereas incisions through the hyponychium may cause the flap to retract and result in significant nail deformity. Meticulous nail bed repair is essential for preventing postoperative nail deformities. Pulp lesions can be approached through a lateral incision.

## COMPLICATIONS

In addition to nail deformity, recurrence is a possible complication and may occur in up to 20% of cases.^2^ Recurrence is thought to be a result of incomplete excision or, in the case of late recurrence, development of a new lesion at or near the excision site. Excision of the capsule of the tumor is required to prevent local recurrence.

## MALIGNANT VARIANT

Glomangiosarcoma is an exceptionally rare malignant variant of the glomus tumor. It tends to appear as a painful nodule located in the subcutaneous tissue. Histology of the glomangiosarcoma tumor shows features that resemble a benign glomus tumor. Nonetheless, malignant glomus tumors arise de novo. This neoplasm is considered a low-grade malignant tumor with tendency for local recurrence, although metastasis has been reported. Treatment consists of complete local excision and close surveillance.^12^

## Figures and Tables

**Figure 1:**
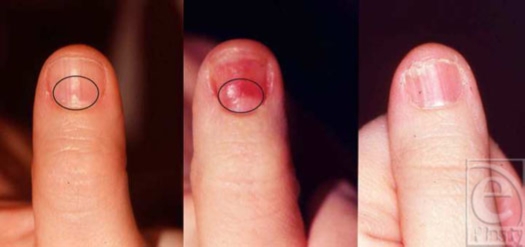
A 30-year-old woman with a subungual glomus tumor of the left thumb. Preoperative view of the perionychium with the lesion identified (left); intraoperative view of the lesion after the nail plate is removed (center); and two-year follow-up demonstrating minimal nail deformity after excision of the tumor and nail bed repair (right).

**Figure 2:**
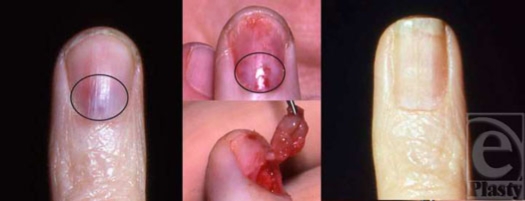
A 44-year-old woman with subungual tumor of the ring finger. Patient presented with 3-month history of nail discoloration and increase in pain and cold sensitivity. Preoperative view of the nail with the lesion identified (left); lesion after removal of the nail plate (center, above); excision of tumor from the nail matrix (center, below); and postoperative view at 2 years demonstrating mild nail deformity (right).
